# Silicon Drift Detectors for the Measurement and Reconstruction of Beta Spectra

**DOI:** 10.3390/s24248202

**Published:** 2024-12-22

**Authors:** Andrea Nava, Leonardo Bernardini, Matteo Biassoni, Tommaso Bradanini, Marco Carminati, Giovanni De Gregorio, Carlo Fiorini, Giulio Gagliardi, Peter Lechner, Riccardo Mancino, Chiara Brofferio

**Affiliations:** 1 Dipartimento di Fisica G. Occhialini, Università Degli Studi di Milano-Bicocca, 20126 Milano, Italy; t.bradanini@campus.unimib.it (T.B.); g.gagliardi5@campus.unimib.it (G.G.); 2 INFN, Sezione di Milano-Bicocca, 20126 Milano, Italy; matteo.biassoni@mib.infn.it; 3 DEIB, Politecnico di Milano, 20133 Milano, Italy; leonardo.bernardini@polimi.it (L.B.); marco1.carminati@polimi.it (M.C.); carlo.fiorini@polimi.it (C.F.); 4 INFN, Sezione di Milano, 20133 Milano, Italy; 5 Dipartimento di Matematica e Fisica, Università Degli Studi della Campania “Luigi Vanvitelli”, 81100 Caserta, Italy; degregorio@na.infn.it; 6 INFN, Sezione di Napoli, 80126 Napoli, Italy; 7 Halbleiterlabor der Max-Planck-Gesellschaft, 85748 Garching, Germany; peter.lechner@hll.mpg.de; 8 Institute of Particle and Nuclear Physics, Faculty of Mathematics and Physics, Charles University, 18000 Prague, Czech Republic; ricky.mancino@gmail.com; 9 Institut für Kernphysik (Theoriezentrum), Fachbereich Physik, Technische Universität Darmstadt, 64298 Darmstadt, Germany; 10 GSI Helmholtzzentrum für Schwerionenforschung, 64291 Darmstadt, Germany

**Keywords:** silicon drift detectors, *β* spectra, GEANT4 simulations

## Abstract

The ASPECT-BET project, or An sdd-SPECTrometer for BETa decay studies, aims to develop a novel technique for the precise measurement of forbidden beta spectra in the 10 keV–1 MeV range. This technique employs a Silicon Drift Detector (SDD) as the main spectrometer with the option of a veto system to reject events exhibiting only partial energy deposition in the SDD. A precise understanding of the spectrometer’s response to electrons is crucial for accurately reconstructing the theoretical shape of the beta spectrum. To compute this response, GEANT4 simulations optimized for low-energy electron interactions are used and validated with a custom-made electron gun. In this article we present the performance of these simulations in reconstructing the electron spectra measured with SDDs of a ^109^Cd monochromatic source, both in vacuum and in air. The allowed beta spectrum of a ^14^C source was also measured and analyzed, proving that this system is suitable for the application in ASPECT-BET.

## 1. Introduction

Nuclear theories are key components in the interpretation of several results in neutrino physics, as in the case of the 0νββ decay search [1] or the reactor oscillation experiments [2]. However, the lack of a single model capable of accurately predicting all experimental observables results in significant nuclear-related systematics.

The predicted shape of the forbidden β energy spectra is highly dependent on the theoretical description and can be an important tool for discriminating between different nuclear models [3]. In addition, measuring different isotopes with the same experimental setup would improve the ability to rule out theories that cannot predict all spectra within the same framework.

To this end, unprecedentedly precise measurements have recently been made for some forbidden β spectra using various technologies. So far, competitive experiments at room temperature have been realized only for beta-active isotopes naturally present in the detector material itself, as in scintillators (e.g., L176u in LSO:Ce or LuAG:Pr [4]) or in semiconductors (as ^113^Cd in CdZnTe [5]). The best results in energy threshold and resolution have been achieved with scintillating low-temperature detectors, such as ${\mathrm{LiInSe}}_{2}$ [6] and LiI [7] for ^115^In, and with metallic magnetic calorimeters (MMCs) used to measure forbidden β spectra of ^36^Cl [8], ^151^Sm [9] and ^99^Tc [10].

MMCs are of particular interest because they can embed the beta-active isotope in the thermal absorber (a very thin gold foil), thus satisfying the requirement to measure different isotopes with the same setup in a relatively simple way [11]. Moreover, they exhibit an excellent energy threshold (O(1) keV) and resolution (O(100) eV at 300 keV) [10]. However, they have all the technical drawbacks of working at very low temperatures with dilution refrigerators. For instance, some isotopes with short half-lives cannot be studied due to the time needed to dilution refrigerators to reach base temperature. In addition, the strict requirement for absorber dimensions also limits the maximum mass of the isotope that can be safely embedded, discouraging the use of MMCs with extremely rare isotopes and/or extremely long half-lives. Embedding the source inside the absorber also implies the need for different absorbers when studying different isotopes.

ASPECT-BET (An sdd-SPECTrometer for BETa decay studies) is a project that aims to measure a set of forbidden β spectra using Silicon Drift Detectors (SDDs) [12]. These devices differ from conventional silicon detectors due to a transverse drift generated by ring electrodes that guide the electrons produced after an energy deposition to a small anode. This layout allows high-rate measurements with an excellent energy resolution (∼200 eV at 5.9 keV). Because of these features, so far the main SDD application has been X-ray spectroscopy [13]. However, these detectors are currently being developed for electron spectroscopy as well, in the context of TRISTAN (TRItium STerile to Active Neutrino investigation), the upgrade of the KATRIN (KArlsruhe TRItium Neutrino experiment) detector to search for keV sterile neutrinos [14].

SDDs are suitable candidates for precise β spectroscopy due to several advantages. As already mentioned, SDDs can operate at room temperature with good energy resolution and can sustain a high interaction rate [15]. Operating at room temperature also allows the measurement of short half-life isotopes, such as those produced in reactors, making this technique complementary to cryogenic techniques. SDDs can be used in conjunction with auxiliary veto detectors, such as scintillators, to reject events where only a fraction of the total energy is deposited in the main detector. Finally, the complete decoupling of the SDD spectrometer from the source simplifies the use of the same setup to study different isotopes.

Spectroscopy of electrons with energies in the 10 keV–1 MeV range from an external radioactive source presents several challenges, the most important being:

- Energy loss in the source, especially if the active material is thick or encapsulated (self-absorption);
- Incomplete charge collection due to a partial charge collection efficiency in the detector entrance window, usually present in all silicon devices;
- Incomplete energy deposition due to electron backscattering after impinging on the detector;
- Incomplete energy deposition due to escape of characteristic X-rays and bremsstrahlung;
- Incomplete charge collection if the interaction is too close to the detector boundaries.

Our goal is to develop a complete model of the system, including both the source and the detector. The electron energy loss in the source and in the detector cannot be accurately predicted starting from the Bethe-Bloch formula, due to the large angle change an electron can experience after a scattering. We then decided to construct a model of the experimental apparatus based on GEANT4 Monte Carlo (MC) simulations [16] and analytical descriptions of detector non-idealities, that can account for all the aforementioned effects in order to accurately predict the shape of electron spectra measured with an SDD.

Some of these parameters, such as the energy loss in the dead layer or the electron backscattering, have already been studied in the KATRIN context [17,18]. Additional measurements performed with a custom-made electron gun are described in this work. A large effort has been put into the construction of a model that accounts for the source-related effects that affect the spectral shape.

We decided to take a stepwise approach, starting with a commercial encapsulated monochromatic electron source measured in vacuum and air. The aim is to test the ability of GEANT4 to simulate low-energy electron scattering on light materials, such as the source plastic capsule and the air, and then to extend the study to a measurement with an allowed β-decay source.

## 2. Experimental Setup

The SDD chip used for this work was built at MPP-HLL (Munich, Germany) using the same design of the TRISTAN detector’s devices, each consisting of a 47-pixel SDD matrix, 450 μm thick. The pixels are hexagonal with sides 1.5 mm long. Since these detectors are intended for spectroscopy of electrons with energies down to 10 keV or less, they have a very thin ${\mathrm{SiO}}_{2}$ entrance window, approximately 10 nm thick.

**Figure 1:**
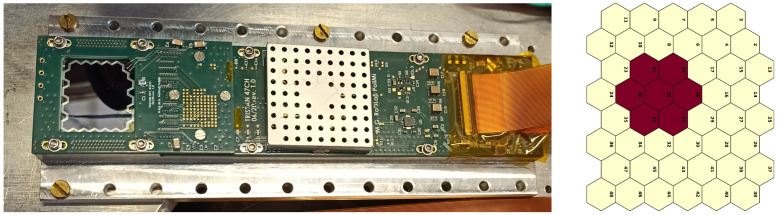
47-pixel SDD matrix used for all the measurements here reported (**left**). Scheme of the 47 pixels: only the 7 red ones were acquired (**right**).

Our SDD chip was operated in a vacuum chamber where a vacuum level down to $10^{-5}$ mbar can be achieved. A 12-channel custom-made ASIC named ETTORE [19] was used as the first level of amplification inside the vacuum chamber, followed by a CAEN DT5743 digitizer located outside, in the vicinity of the chamber to keep the connections as short as possible. For this work, we acquired a group of 7 adjacent SDDs, our “SDD flower”, as shown in Figure 1. Their choice, within the 47 pixels of the SDD chip, was guided by their similar energy resolutions and thresholds. Among these 7 SDDs, the central one was acquired as the main pixel, while the surrounding 6 were used as veto, to reject those events with an energy deposition shared between one of them and the main one (M2, or “multiplicity-2 events”).

For each triggered event, the 7 signals were digitized and captured at the waveform level, resulting in 7 waveforms, each 2.5 μs long. A digital trapezoidal filter was applied offline to extract an accurate estimate of the amplitude. The M2 events were identified by looking at the number of channels with an amplitude greater than a given threshold. Using only the events with multiplicity one (M1) the energy spectrum was constructed.

Once the data acquisition system was completed, the SDD flower was calibrated in energy using X-rays from a ^55^Fe and a ^109^Cd source.

## 3. Characterization of the SDD Response to External Electrons

In order to perform and understand β-spectra measurements, the SDD response to external electrons must be investigated. Radioactive electron sources are not ideal for these measurements due to self-absorption; this effect has to be considered in simulations, and it is also correlated with the detector response itself making it difficult to distinguish. To overcome this limitation, we have developed a photoelectric-based electron gun (e-gun) capable of providing a monochromatic and mono-angular electron beam. Such a source is easy to simulate and therefore ideal for testing the detector response.

**Figure 2:**
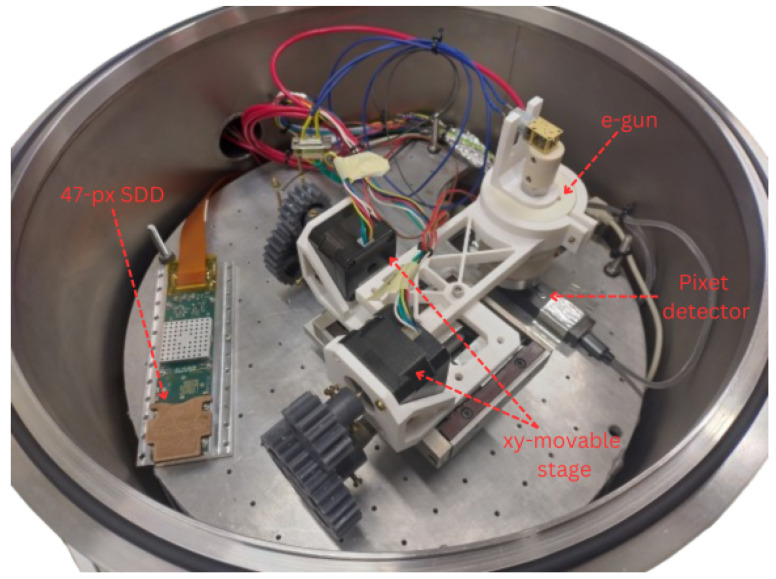
Vacuum chamber in the Milano-Bicocca laboratory. The main detectors operated in this setup, a 47-pixel SDD matrix and a Pixet, are indicated. The e-gun, attached to a xy-movable stage, is also highlighted.

In order to avoid discharges, the e-gun must operate in a vacuum. The vacuum chamber that has been constructed in the laboratory of Milano-Bicocca, used for all the measurements described in this work, is shown in Figure 2. The main devices operated in the vacuum chamber are highlighted. 

The two detectors operated in this chamber are:

- the 47-pixel SDD matrix;
- a Pixet detector [20], which is a multipixel (256 × 256 pixels) silicon detector providing excellent time and spatial resolution.

**Figure 3:**
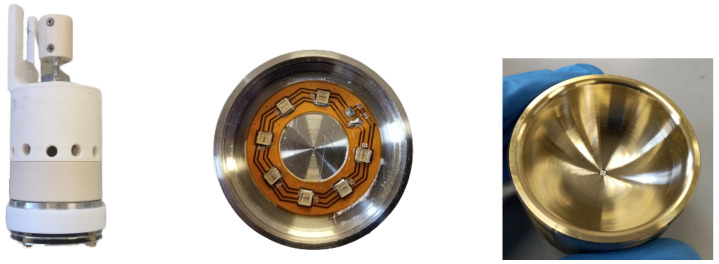
(**Left**): picture of the e-gun. (**Center**): anode with 7 LEDs used to illuminate the cathode and the aluminum cylinder with UV light. (**Right**): gold-coated cathode used to collimate the electron beam. The aluminum cylinder, where electrons are produced, is visible in its center.

**Figure 4:**
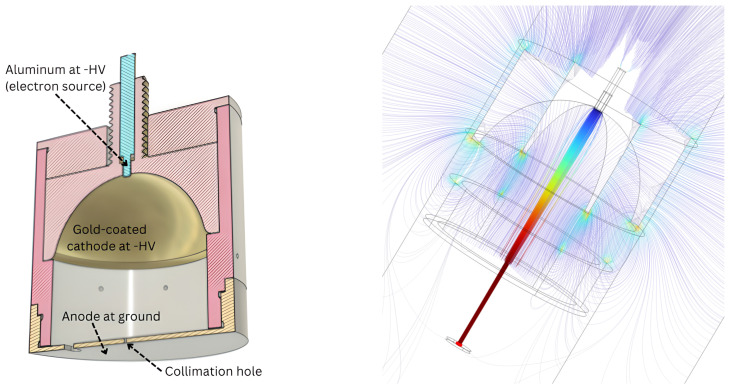
(**Left**): CAD drawing of the e-gun. The most important components are highlighted. (**Right**): COMSOL simulation of the electron beam produced with the e-gun.

The electron gun is mounted on a motorized xy-movable stage, which is used to accurately point the electron beam to a precise coordinate. For example, it can be set to point to the central part of an SDD, to avoid M2 events, or it can be used to shoot electrons both on the SDD and on the Pixet in the same measurement, without opening the vacuum chamber. A picture of the electron gun is shown in the left panel of Figure 3. The internal structure of the e-gun is instead shown in the CAD in Figure 4. The main components are indicated.

The e-gun working principle is described in the following:

- a group of 7 LEDs (shown in the central picture of Figure 3) are mounted on the anode (which is grounded) and used to produce UV light. In particular, the light emission peaks at a wavelength of 275 nm, roughly corresponding to a photon energy of 4.5 eV. All the internal surfaces of the e-gun are illuminated.
- The cathode, set at negative high voltage (down to −15 kV), is a gold-coated semispheric surface, shown on the left picture of Figure 3. The gold work function is 5.10–5.47 eV, meaning that the production of electrons from the cathode surface is minimal. At the center of the cathode, there is an aluminum cylinder. The aluminum work function is 4.06–4.26. Electrons can be produced by photoelectric effect from this surface. When exiting the aluminum, their kinetic energy is less than 1 eV.
- The produced electrons are accelerated towards the anode thanks to the negative high voltage on the cathode. Its semispherical shape also helps in collimating the electron beam towards a 0.5 mm hole in the anode. Electrons exiting the e-gun have a kinetic energy equal to the high voltage applied to the cathode. A COMSOL [21] simulation of the e-gun beam is shown in Figure 4. This simulation proves the working principle of this electron source and predicts a beam spot size of ∼0.5 mm at the detector position.

**Figure 5:**
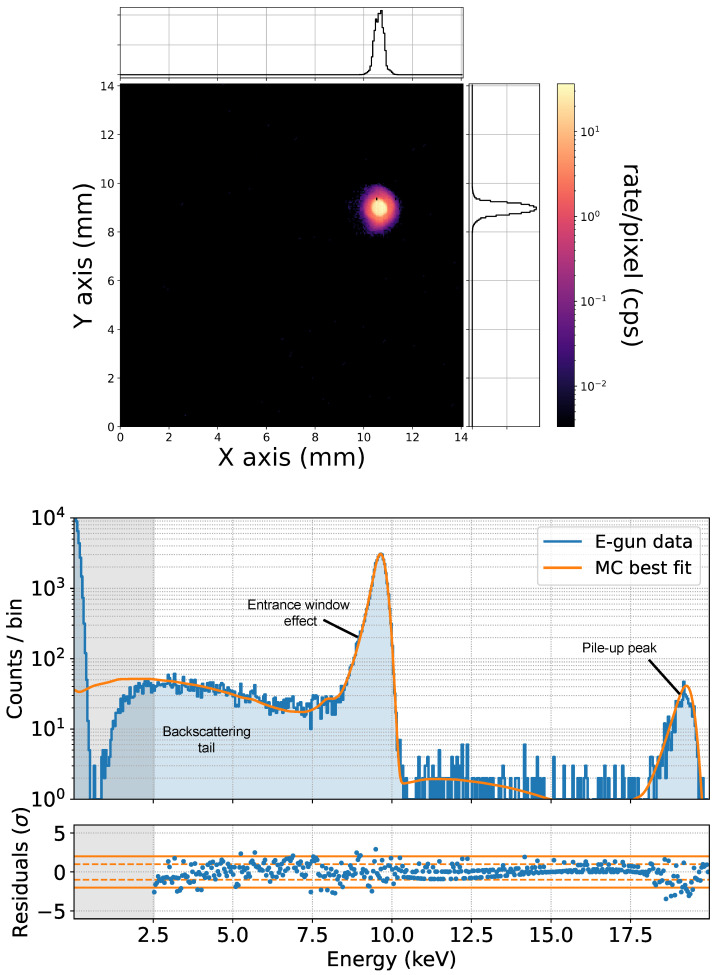
(**Top**): measurement of the e-gun beam spot performed with the Pixet. A beam size of ∼0.5 mm and a rate $10^{4}$ electrons/s were found. (**Bottom**): measurement of a 10 keV electron spectrum acquired with an SDD. Only the central part of the pixel was hit with the e-gun beam. The best fit of the spectrum done with the detector model is also shown.

The e-gun aluminum cylinder can be moved inside and outside of the gold cathode. Several measurements were carried out to find the best position of the cylinder in order to have a focused beam and a high electron rate. These measurements were performed with the Pixet detector, exploiting its excellent time and spatial resolution. The plot of the beam spot measured with the Pixet in the optimal cylinder configuration is shown in the left panel of Figure 5. A spot size of ∼0.5 mm, as predicted from the COMSOL simulation, has been found. The electron rate is about $10^{4}$ electrons/s.

Once the e-gun configuration has been optimized with Pixet measurements, its electron beam can be used to study the SDD response. Data acquired with the SDD for an electron energy of 10 keV are shown in the right panel of Figure 5. The measurement was performed by shooting with the e-gun only on the central part of an SDD.

Four main features can be seen in the spectrum.

- A main peak close to the beam energy. The peak is not centered at 10 keV due to the partial charge collection efficiency in the detector entrance window. This effect also causes the asymmetry of the peak: electrons that deposit more energy in the entrance window populate the left tail of the peak. The right tail is instead Gaussian since it’s only determined by the energy resolution of the system.
- A noise peak centered at zero and the energy threshold being approximately 2 keV.
- A tail going from the main peak down to the energy threshold. It is due to those electrons that, after one or more scatterings in silicon, flip the direction and leave the detector. These are called backscattering events.
- A peak due to pile-up at roughly twice the beam energy. This is due to those cases where two electrons deposit energy in the SDD close enough in time that the detection system only identifies one event, in which the energy estimation is the sum of the two energy depositions.

Based on previous studies [18,22], we developed a GEANT4 simulation using the Penelope physics list [23], a package optimized for low-energy electromagnetic physics. The main SDD is simulated as a hexagonal prism surrounded by six other SDDs, and its entrance window is segmented into 30 layers, following the procedure described in [24] to apply depth-dependent charge collection efficiency effects. Backscattering, which only depends on the scattering of an electron with the material, is automatically taken into account in the GEANT4 simulation. The energy resolution is then applied by folding the spectrum with a Gaussian, which width is proportional to the square root of the energy. Concerning pile-up, it is added through a convolution of the spectrum containing all the aforementioned effects with itself. The Monte Carlo reconstruction of the measured spectrum is shown in orange in Figure 5. It can be seen how all the main structures are well reproduced from the simulation. This model is used to take into account the detector response for all the measurements presented in the following sections.

## 4. Measurements with a ^109^Cd Commercial Source

For the first measurements, we used the same commercial ^109^Cd source used for calibration. This source decays by electron capture (EC) to the ^109*m*^Ag metastable state at 88 keV with a half-life of 462.1 days and emits mainly low energy X-rays at ∼3 keV, ∼22 keV and ∼25 keV. The ^109*m*^Ag decays to the ground state mainly by internal conversion (IC) (B.R. ∼96.3%) and has a half-life of 39.6 s. The IC electrons are emitted with different energies depending on the atomic shell they come from (see Table 1).

The active source is deposited in a 3 mm dia. spot and encapsulated between two thin aluminized Mylar foils (each ∼6.5 μm thick, as declared by the producer).

**Figure 6:**
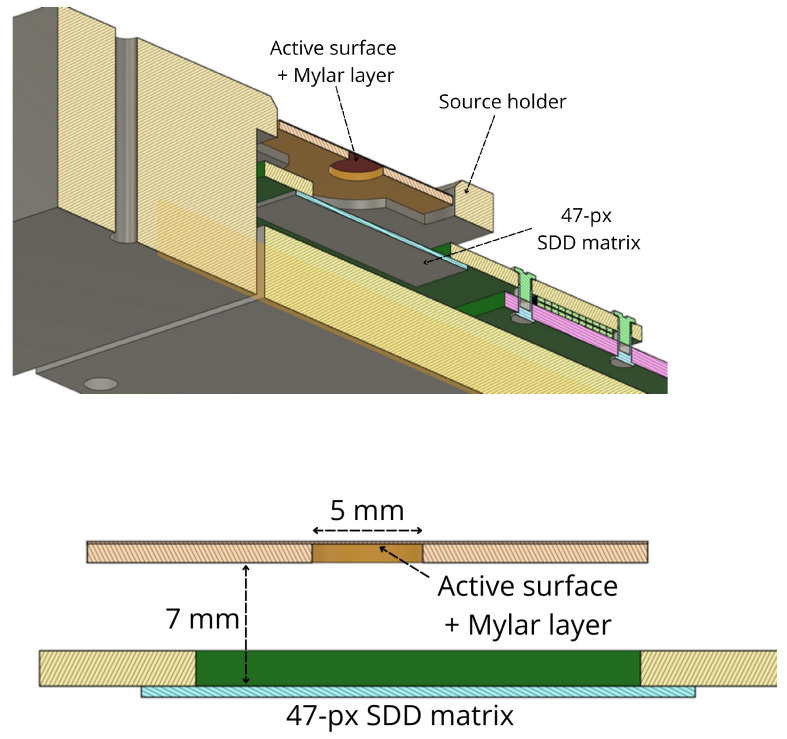
CAD schematics of the setup used in the measurements. Section of source and detector (**top**), and side view (**bottom**).

Due to the energy loss in the Mylar foils, the electrons leaving the source are already less energetic and non-monochromatic. The position of the electron peaks in the energy spectrum and their width in the collected energy spectrum depend on the thickness of the Mylar foils, the presence of air between the source and the detector, and the position of the source with respect to the SDD. To remove one degree of freedom from the simulation, a source holder was 3D printed to center the source with the SDD flower at a fixed distance of 7 mm (as shown in the CAD Figure 6).

**Figure 7:**
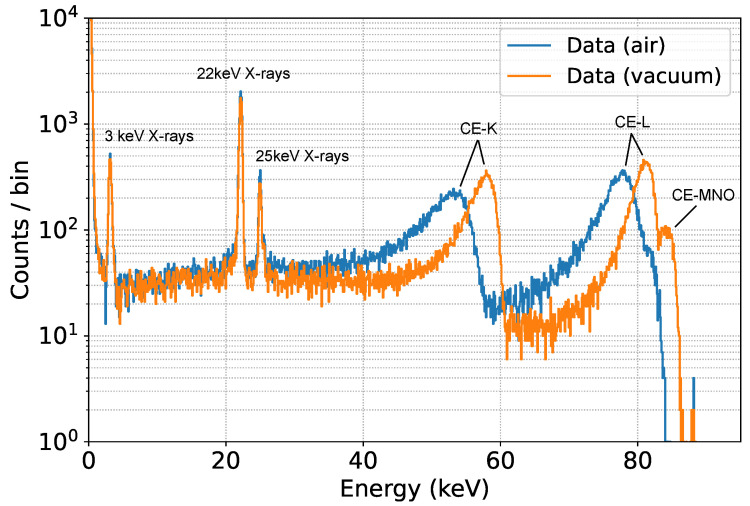
M1 Data acquired with the SDD main pixel in a 3-h measurement. The energy of the X-ray peaks and the tag of the different IC electrons are shown.

Two measurements of 3 h each were performed, one in vacuum at a pressure of $10^{-5}$ mbar and one at atmospheric pressure. The two spectra are shown in Figure 7.

The position of the X-ray peaks is the same in both vacuum and air, as expected, because photons interact directly in the detector, while the electron peaks are shifted to lower energies, depending on the minimum energy loss on their way from the source to the SDD flower, which is higher in the presence of air. Scattering in air also leads to a broader peak.

Our goal is to build a realistic model that can reproduce the shape of these spectra, and therefore the source and the detector were simulated in GEANT4. The model shown in Section 3 is used to take into account the detector response.

It’s worth noting that even with the M2 cut, a small effect of partial charge collection at the SDD boundaries remains. This is due to those events that produce a signal below the threshold in surrounding SDDs, resulting in spurious M1 events. This effect is included in the model and depends on the charge cloud produced in silicon. We assumed the cloud to be Gaussian, with an energy-independent size fixed at 15 μm from previous characterizations [25].

In our GEANT4 model, electrons are generated isotropically between the two Mylar foils. To compare the simulation with the data, we expect the largest systematic effect to be the scattering in the Mylar foils, since the thickness of this part is ∼500 times larger than the detector entrance window. In addition, the peak broadening caused by multi-scattering in the source is clearly larger than the energy resolution of the SDD. Therefore, we decided to fix the entrance window parameters and the energy resolution to values obtained from the characterization measurement shown in Figure 5.

We ran simulations for different thicknesses of these layers, to compensate for other non-simulated materials, such as the small amount of Al in the aluminized Mylar foils or the very thin adhesive layers used in the source fabrication, but whose thickness is not specified. Thus, the main free parameter we use is an effective thickness of the Mylar foils. The best-fit estimate for the effective Mylar thickness is the one that minimizes the $\chi^{2}$ between the MC prediction and the data. Since the measured electrons are at higher energies than the X-rays used to calibrate, two additional nuisance parameters (a horizontal gain and shift) are left free in the $\chi^{2}$ minimization. For all the fits the best estimation for these parameters is compatible with the calibration made using X-rays.

**Figure 8:**
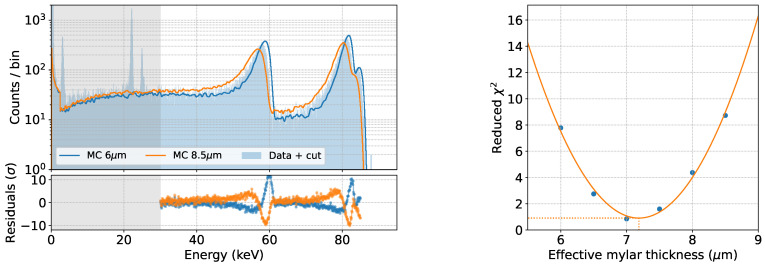
Data-MC comparison for different values of the effective Mylar thickness (**top**). Reduced $\chi^{2}$ as a function of the effective Mylar thickness (**bottom**). The best-fit value of 7.2 μm is extracted through a parabolic fit.

Figure 8 shows the simulated spectra assuming two different values of the effective Mylar thickness: 6 μm and 8.5 μm. A larger (smaller) thickness is reflected in a larger (smaller) shift of the ^109^Cd IC electron peaks toward lower energies and on a broader (narrower) shape of the peaks themselves.

**Figure 9:**
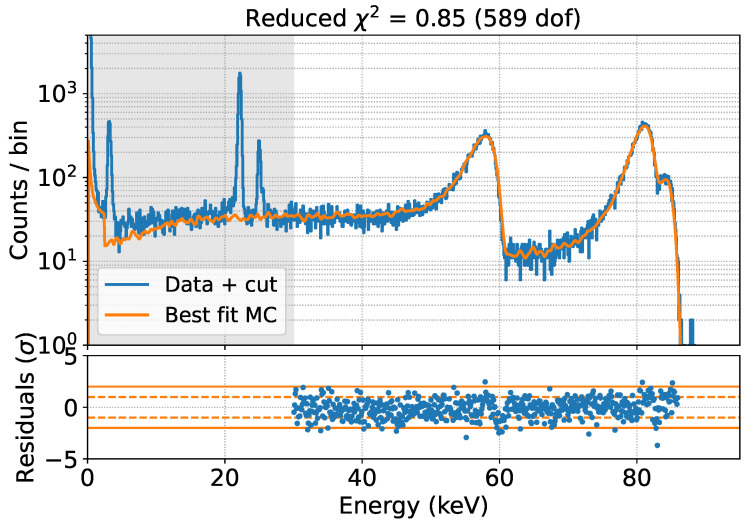
Best fit of MC prediction to the data set acquired with the ^109^Cd source in vacuum. The fit is done only in the non-shaded area.

The data-MC comparison is performed in the energy region >30 keV to avoid contributions from non-simulated Ag X-rays, for five different values of the effective Mylar thickness. A parabolic fit of the $\chi^{2}$ as a function of the effective Mylar thickness yields a best-fit estimate of 7.2 μm for this parameter, as shown in Figure 8. The corresponding spectrum is shown in Figure 9.

The fit result is in excellent agreement with the data, and in particular, all major structures in the spectrum are well reproduced, from the positions of the peaks to their widths and the shape of their tails.

**Figure 10:**
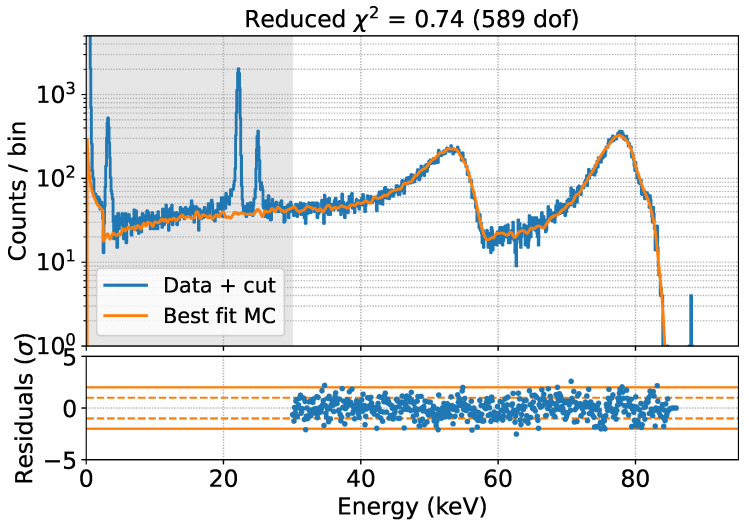
Comparison of MC prediction to the dataset acquired with the ^109^Cd source in air. The comparison is done only in the non-shaded area.

After this step, we decided to fix the best-fit result for the effective Mylar thickness and try to reproduce the air data set without adding any new free parameters, as a cross-check of GEANT4’s ability to predict the effect of additional material interposed between the source and the detector. The comparison is shown in Figure 10.

The excellent reproduction of the data with our GEANT4 model demonstrates the reliability of the model and its robustness to changes in the experimental conditions.

## 5. Measurements with ^14^C

We then switched to a commercial ^14^C source encapsulated between a 100 μm thick paper foil on the back and a thin aluminized Mylar layer on the front.

The ^14^C allowed β decay has a Q-value of ∼156 keV and an average electron energy of ∼50 keV. So we pushed our measurements to slightly higher electron energies.

**Figure 11:**
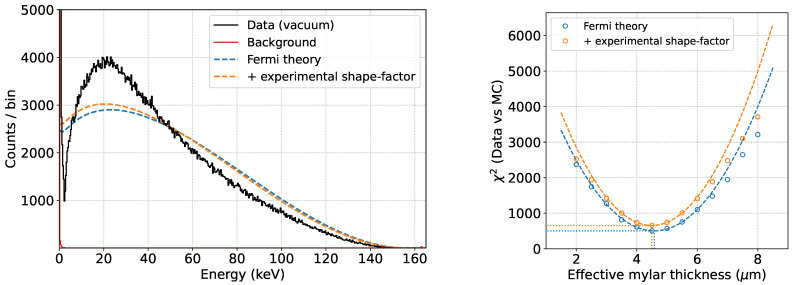
Data acquired with the main SDD in a 6 h measurement using a ^14^C source (**top**). $\chi^{2}$ as a function of the effective Mylar thickness for the two models: the Fermi theory prediction and the one including the experimental shape factor (**bottom**). The best fit for the effective Mylar thickness is 4.5 μm.

We performed a 6 h measurement using the same setup described in the previous section. The resulting spectrum, calibrated against the ^109^Cd peaks, is shown in Figure 11.

The theoretical spectral shape of C14 can be obtained using a software such as Betashape [26]. In particular, two spectra are available: a purely theoretical prediction based on β kinematics and the Fermi factor, and a prediction including an energy-dependent experimental shape factor measured in [27]. The shapes of the two predictions, normalized to the integral above 15 keV, are shown in Figure 11 with a blue and an orange dashed line respectively. These spectra were used as input for the GEANT4 simulations.

It is clear how the response of the system changes the shape of the theoretical spectrum, again highlighting the need for an accurate and reliable simulation to interpret the experimental measurements.

We performed the same analysis as for the ^109^Cd case for these two models, varying the effective Mylar thickness in the simulation. The result of the fit, performed starting at 15 keV, is shown in Figure 11.

**Figure 12:**
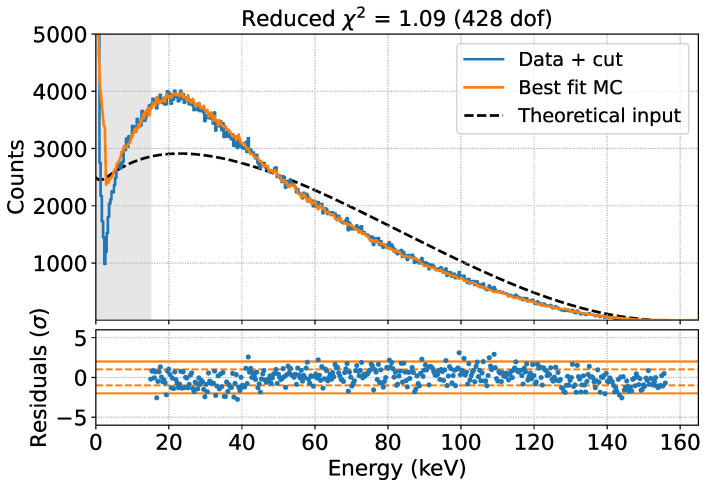
Fit to the data set acquired with the ^14^C source in vacuum using the MC prediction. The fit is performed only in the non-shaded area. The theoretical input is also shown.

In both cases, the best fit value for the effective Mylar thickness is 4.5 μm, indicating that the effect of this thickness is not degenerate with the shape factor. The pure Fermi model, without the shape factor, better describes the data for all values of the only free parameter (the effective Mylar thickness), with a $\chi^{2}$ difference between the two models of about 150 at their minimum, indicating that Fermi theory is preferred to explain the ^14^C spectrum. The best fit, without any experimental shape factor, is shown in Figure 12.

**Figure 13:**
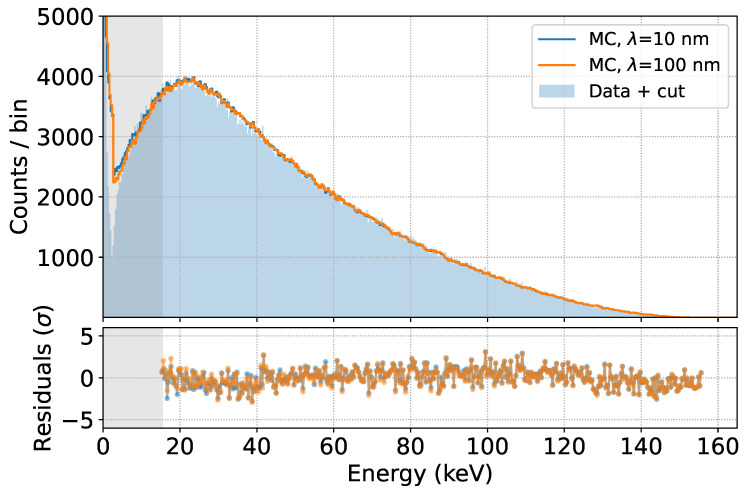

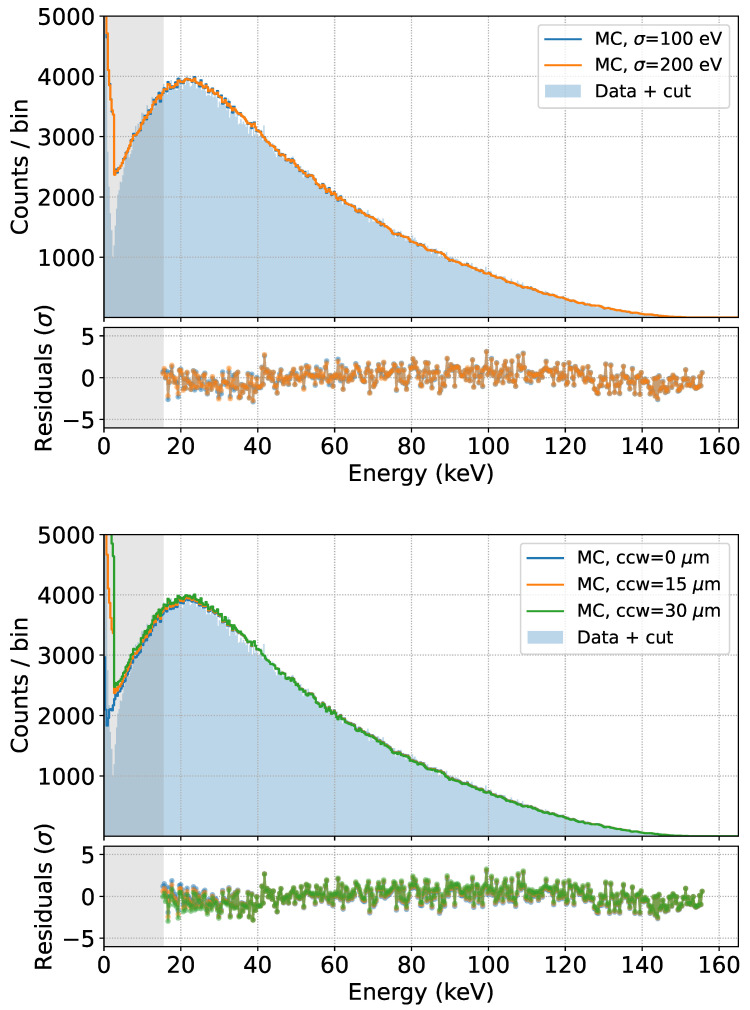
Fits obtained by varying λ (**top**), the baseline resolution σ (**center**) and the charge cloud width in Si (**bottom**).

We then tested the robustness of the model’s prediction by comparing the different simulation results obtained by varying the parameters of the detector response, namely the λ parameter related to the depth-dependent charge collection efficiency (default: λ = 55 nm [18]), the baseline energy resolution σ (default: σ = 150 eV), and the charge cloud width (ccw) in silicon, which mimics the partial charge collection at the SDD border (default: ccw = 15 μm). The results are shown in Figure 13.

The effect on the fit quality of using a ten times larger λ parameter is negligible, as is the effect of using a resolution 50 eV higher or lower than the reference. This is again due to the fact that the scattering within the source dominates the broadening of the measured spectra. The effect of a zero or doubled charge cloud with respect to the standard results in only a small change in the low energy part of the spectrum, where the multiplicity cut is less efficient. We can therefore conclude that the spectrum prediction above 15 keV is almost independent of the parameters of the SDD response.

**Figure 14:**
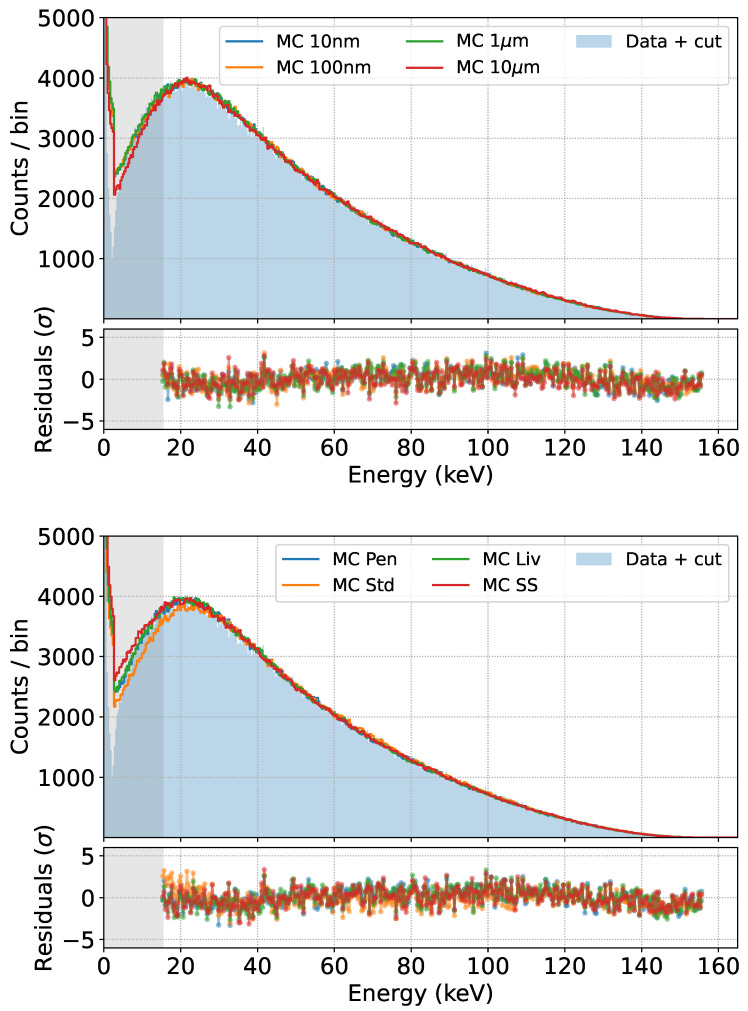
Fits obtained by varying the production cut for secondaries in GEANT4 (**top**) or the physics list used (**bottom**) in the simulations.

To further assess the robustness of our prediction, we also repeated the comparison by varying some settings of the GEANT4 simulation, namely the secondary production cut (the distance that secondary particles have to travel in a given material to be produced in GEANT4) and the physics list chosen (the set of physical models used in the simulation) [28]. The results are shown in Figure 14.

We observe that an effect appears only in the low energy region, and only for production cuts above 10 μm, while the default best value for our simulations is 10 nm.

Regarding the available physics lists, only the Standard Electromagnetic one [28] produces worse results in the low energy region of the spectrum, while the physics lists specifically designed for low energy electromagnetic interactions, such as Penelope, Livermore, and Single Scattering [28], produce the same result in the region of interest.

## 6. Conclusions and Outlook

In this work we have demonstrated the ability of SDDs to measure electrons in the 15–150 keV range using commercial ^109^Cd and ^14^C sources. This represents the first application of SDDs as electron spectrometers at energies approximately one order of magnitude higher than those typically involved in measurements with these devices. It is furthermore a fundamental step towards the ASPECT-BET goal of performing β spectroscopy in the 10 keV–1 MeV energy range. The need for an accurate model of the system response has been emphasized, and in this context, the performance of GEANT4 low-energy simulations in reconstructing the measured spectra has also been shown. The parameters of the simulation and the knowledge of the detector response are of minor importance as long as we use sources encapsulated in thin passive layers. Instead, the most important systematic effect is the exact knowledge of the thickness of this layer. By varying the effective thickness, we were able to obtain an excellent reconstruction of the shape of the ^109^Cd monochromatic lines, both in vacuum and in air, and of the ^14^C β spectrum. Other measurements found in the literature [27] required the introduction of an experimental shape factor to explain the shape of the measured spectrum, while in our case this requirement is rejected with high significance.

With these measurements, we have therefore validated a new SDD-based technique for measuring β spectra, which is important for its complementarity with respect to cryogenic calorimetric measurements and for its versatility in source selection.

In the near future we will switch to a new detector technology using larger and thicker SDDs. The side length will be ∼1 cm, leading to a smaller fraction of events near the borders, and therefore to an even smaller systematics related to partial charge collection. The thickness will be 1 mm, allowing us to measure electrons with higher energies that would otherwise not be fully contained in the detector. The first decay we will study is the non-unique second forbidden decay from a commercial ^99^Tc source, which has been recently measured using MMCs, and whose shape was found to be sensitive to different assumptions made in nuclear theory calculations [10].

We are also planning to improve the source-related systematics by actually depositing the radioactive material on an auxiliary detector, to avoid a passive layer between the source and the main SDD, and to allow anti-coincidence measurements, by which those events with only partial energy deposition in the main SDD can be vetoed.

A final consideration concerns the possibility of studying the half-lives of isotopes: in this work, we have carried out a shape-only analysis of the spectra, but when switching to custom deposited sources, through a precise knowledge of the amount of the isotope of interest, half-life measurements will become possible, although still challenging.

## Figures and Tables

**Table 1:** Table of ^109^Cd electron lines.

Shell	Electron Energy (keV)	Intensity (%)
K	62.5	41.8
L	∼84.4	44.1
M-N-O	87–88	10.4

## Data Availability

The raw data supporting the conclusions of this article will be made available by the authors on request.
